# Effectiveness and safety of rituximab in severely relapsed antineutrophil cytoplasmic antibody–associated vasculitis: a retrospective analysis of a Japanese multicentre cohort from the J-CANVAS

**DOI:** 10.1007/s10067-024-07096-y

**Published:** 2024-08-12

**Authors:** Genki Kidoguchi, Yusuke Yoshida, Hirofumi Watanabe, Tomohiro Sugimoto, Sho Mokuda, Takashi Kida, Nobuyuki Yajima, Satoshi Omura, Daiki Nakagomi, Yoshiyuki Abe, Masatoshi Kadoya, Naoho Takizawa, Atsushi Nomura, Yuji Kukida, Naoya Kondo, Yasuhiko Yamano, Takuya Yanagida, Koji Endo, Kiyoshi Matsui, Tohru Takeuchi, Kunihiro Ichinose, Masaru Kato, Ryo Yanai, Yusuke Matsuo, Yasuhiro Shimojima, Ryo Nishioka, Ryota Okazaki, Tomoaki Takata, Takafumi Ito, Mayuko Moriyama, Ayuko Takatani, Yoshia Miyawaki, Toshiko Ito-Ihara, Takashi Kawaguchi, Yutaka Kawahito, Shintaro Hirata, on behalf of Japan Collaborative Registry of ANCA-Associated Vasculitis (J-CANVAS)

**Affiliations:** 1 Department of Clinical Immunology and Rheumatology, Hiroshima University Hospital, Hiroshima, Japan; 2 Inflammation and Immunology, Graduate School of Medical Science, Kyoto Prefectural University of Medicine, Kyoto, Japan; 3 Division of Rheumatology, Department of Medicine, Showa University School of Medicine, Tokyo, Japan; 4 Department of Rheumatology, University of Yamanashi Hospital, Yamanashi, Japan; 5 Department of Internal Medicine and Rheumatology, Juntendo University, Tokyo, Japan; 6 Center for Rheumatic Disease, Japanese Red Cross Society Kyoto Daiichi Hospital, Kyoto, Japan; 7 Department of Rheumatology, Chubu Rosai Hospital, Aichi, Japan; 8 Immuno-Rheumatology Center, St. Luke’s International Hospital, Tokyo, Japan; 9 Department of Rheumatology, Japanese Red Cross Society Kyoto Daini Hospital, Kyoto, Japan; 10 Department of Nephrology, Kyoto Katsura Hospital, Kyoto, Japan; 11 Department of Respiratory Medicine and Allergy, Tosei General Hospital, Aichi, Japan; 12 Department of Hematology and Rheumatology, Kagoshima University Hospital, Kagoshima, Japan; 13 Department of General Internal Medicine, Tottori Prefectural Central Hospital, Tottori, Japan; 14 Department of Pharmacoepidemiology, Graduate School of Medicine and Public Health, Kyoto University, Kyoto, Japan; 15 Department of Diabetes, Endocrinology and Clinical Immunology, School of Medicine, Hyogo Medical University, Hyogo, Japan; 16 Department of Internal Medicine (IV), Osaka Medical and Pharmaceutical University, Osaka, Japan; 17 Department of Immunology and Rheumatology, Division of Advanced Preventive Medical Sciences, Nagasaki University, Nagasaki, Japan; 18 Department of Rheumatology, Faculty of Medicine, Shimane University, Shimane, Japan; 19 Department of Rheumatology, Endocrinology and Nephrology, Graduate School of Medicine, Hokkaido University, Hokkaido, Japan; 20 Department of Rheumatology, Tokyo Kyosai Hospital, Tokyo, Japan; 21 Department of Rheumatology, Graduate School of Medical and Dental Sciences, Tokyo Medical and Dental University (TMDU), Tokyo, Japan; 22 Department of Medicine (Neurology and Rheumatology), Shinshu University School of Medicine, Nagano, Japan; 23 Department of Rheumatology, Graduate School of Medical Science, Kanazawa University, Ishikawa, Japan; 24 Division of Respiratory Medicine and Rheumatology, Department of Multidisciplinary Internal Medicine, Faculty of Medicine, Tottori University, Tottori, Japan; 25 Division of Gastroenterology and Nephrology, Tottori University, Tottori, Japan; 26 Division of Nephrology, Department of Internal Medicine, Teikyo University Chiba Medical Center, Chiba, Japan; 27 Rheumatic Disease Center, Sasebo Chuo Hospital, Nagasaki, Japan; 28 Department of Nephrology, Rheumatology, Endocrinology and Metabolism, Dentistry and Pharmaceutical Sciences, Okayama University Graduate School of Medicine, Okayama, Japan; 29 The Clinical and Translational Research Center, University Hospital, Kyoto Prefectural University of Medicine, Kyoto, Japan; 30 Department of Practical Pharmacy, Tokyo University of Pharmacy and Life Sciences, Tokyo, Japan

**Keywords:** ANCA-associated vasculitis, Granulomatosis with polyangiitis, Microscopic polyangiitis, Relapse, Rituximab

## Abstract

We aimed to clarify the long-term safety and efficacy of rituximab (RTX) as a remission induction therapy following severe relapse in patients with antineutrophil cytoplasmic antibody (ANCA)–associated vasculitis (AAV). We retrospectively collected the data of patients with severely relapsed AAV from a Japanese multicentre cohort. The primary exposure was RTX use; the primary outcome was complete remission (CR) proportions at week 24. Baseline characteristics were compared between the RTX and non-RTX groups. We performed multivariate logistic regression analysis and one-to-one propensity score matching analysis as a sensitivity analysis. Totally, 100 patients were enrolled: 52 in the RTX group and 48 in the non-RTX group. Baseline characteristics were comparable between the two groups, except for age, AAV subtype and ANCA serotype. The median age was 71 vs. 75 years, and the PR3-ANCA positivity rate was 44.2% vs. 18.8% in the RTX and non-RTX groups, respectively. No significant difference was observed in CR proportions at week 24 between the two groups (79.2% vs. 68.1%, *p* = 0.321), with an adjusted odds ratio of 1.27 (95% confidence interval [CI] 0.47–3.51). At week 48, CR proportions were significantly higher in the RTX group (91.7% vs. 64.9%, *p* = 0.005), with an adjusted odds ratio of 2.95 (95% CI 0.97–9.91). Serious infection rates were lower in the RTX group than in the non-RTX group, with no statistically significant difference. RTX was not superior to conventional immunosuppressive therapies at week 24 but showed significantly favourable results at week 48 for severely relapsed AAV.

**Supplementary Information:**

The online version contains supplementary material available at 10.1007/s10067-024-07096-y.

**Table:** 

**Key Points**
• *RTX might be superior to traditional treatments as an induction therapy for severely relapsed AAV.* • *RTX has the potential to sustain long-term remission with fewer occurrences of infections in the treatment of severely relapsed AAV.*

## Introduction

Antineutrophil cytoplasmic antibody (ANCA)–associated vasculitis (AAV) is a severe systemic small-vessel disease characterized by the presence of autoantibodies such as autoantibodies against neutrophil proteins, leukocyte proteinase 3 (PR3-ANCA) and myeloperoxidase (MPO-ANCA) and categorized into granulomatosis with polyangiitis (GPA), microscopic polyangiitis (MPA) and eosinophilic GPA (EGPA) based on clinical features [1].

Recently, early diagnosis followed by prompt treatment improved the 5-year survival rate of patients with AAV. However, high relapse rates after remission remain a concern. Depending on the treatment, relapse rates of 21–89% have been reported to occur 5 years after the initiation of induction therapies [2]. Another review reported that one-third of the patients with recurrence had a severe disease [1].

Historically, cyclophosphamide has been commonly used in remission induction therapy for severe and relapsed cases. However, the side effects associated with cumulative doses of cyclophosphamide have become problematic, leading to a shift toward rituximab (RTX) as the primary therapy. RTX, an anti-CD20 monoclonal antibody, plays a key role in AAV treatment by depleting B cells [3–5]. RTX has been shown to be effective in treating AAV, with remission rates comparable to that of cyclophosphamide [6]. In addition, RTX has demonstrated a tolerable safety profile across diverse age groups [7–9]. Recent studies have reported promising results regarding the effectiveness of RTX in relapsed AAV [10–12].

However, recent studies assessing the effects of RTX on recurrent AAV, which included patients from the RITAZAREM and RAVE trials, wherein some relapses were mild, have predominantly focused on short-term outcomes [10, 11]. Another recent study from Japan compared the effectiveness and safety of RTX and intravenous cyclophosphamide for the treatment of life-threatening AAV [13]. However, the limitations of this study are that it focused on short-term outcomes over a 60-day period and did not distinguish between new-onset and recurrent cases. Moreover, because it was based on data extracted from a nationwide inpatient database in Japan, detailed information on disease activity, diagnostic validity, laboratory data and therapeutic agents was unavailable. Therefore, a more comprehensive exploration of RTX use in severe relapse scenarios is required. Current studies leave a significant gap in the understanding of the long-term effectiveness and safety of RTX as a remission induction therapy after severe relapse in real-world settings.

Therefore, we examined the effectiveness of RTX in treating patients with relapsed AAV in Japan, using multicentre registry data. This study is pivotal for improving the care of patients with AAV in Japan and other regions.

## Materials and methods

### Study design

We conducted a multicentre retrospective cohort analysis using data from the Japan Collaborative Registry of ANCA-Associated Vasculitis (J-CANVAS), a multicentre registry established by 24 referral sites in Japan.

### Setting

The registry enrolled adult patients (aged ≥ 20 years) who were newly diagnosed with AAV or experienced a relapse between January 2017 and June 2020. All patients were classified as having MPA, GPA or EGPA based on the definitions of the 2012 International Chapel Hill Consensus Conference and European Medicines Agency algorithm [14]. The duration of follow-up for each patient ranged from the onset of disease to the occurrence of mortality, loss to follow-up or June 2021.

### Participants

This study included patients with MPA and GPA who had achieved remission (Birmingham Vasculitis Activity Score [BVAS] = 0) and subsequently experienced severe relapses, defined as life- or organ-threatening, to determine the effectiveness of RTX [15].

### Data collection

We retrospectively collected clinical information from the clinical records of each medical site. Baseline characteristics were collected before initiating or enhancing treatment, including patient demographics (age, sex, AAV subtypes [MPA/GPA/EGPA] and ANCA serotypes [MPO-ANCA/PR3-ANCA]). Additionally, data on various factors were collected, including the BVAS 3.0 at relapse [16], medication received at relapse (such as glucocorticoids and immunosuppressants), achievement of complete remission (CR) induction at 24 and 48 weeks after relapse, treatment with concomitant immunosuppressants during the follow-up period and the incidence of severe infections.

### Exposures

Primary exposure was defined as the administration of RTX at least once after relapse, categorizing the administration frequency as one, two, three or four times. The dose and frequency of RTX were determined by each clinician.

### Outcomes

The primary outcome was the proportion of patients achieving CR at 24 weeks. CR was defined as a BVAS of 0, irrespective of the use of immunosuppressive drugs. Secondary outcomes were the proportion of patients with CR at 48 weeks, BVAS at 24 and 48 weeks, severe infections occurring within the 48-week period and glucocorticoid dosage during the follow-up period.

### Statistical analysis

Age, glucocorticoid dosage and BVAS were the continuous variables. Sex, ANCA serotype, AAV subtype, concurrent medication, achievement of CR and occurrence of serious infection were the categorical variables. Summary statistics were presented as median values and quartile ranges or as numbers with proportions. We conducted univariate analysis of the variables between the RTX and non-RTX groups. Continuous variables were analysed using the Wilcoxon rank sum test, and categorical variables were analysed using the chi-square test.

We conducted logistic multivariate analysis to estimate the odds ratio of achieving CR with RTX use. We selected sex, AAV subtype, ANCA serotype and glucocorticoid dosage at reinduction treatment as covariates because of their clinical relevance in achieving CR in patients with AAV, as previously reported [17, 18]. We conducted subgroup analyses to explore the interactions of RTX with ANCA serotype and AAV subtype.

We assumed that collected data were randomly missing. Consequently, multiple imputations were performed for outcomes used in multivariate analysis, and 50 imputed datasets were created. We defined cases of death at 24 and 48 weeks as non-remission.

In the sensitivity analysis, we performed a propensity score matching analysis between the RTX and non-RTX groups. We used a logistic regression model to estimate the propensity scores and applied the nearest neighbour matching with a calliper of 0.25. The matching ratio was 1:1 based on age, AAV subtype, ANCA serotype, initial glucocorticoid dosage and initial BVAS score at relapse. Additionally, we conducted a complete case analysis. Finally, we confined the control group to a non-RTX cohort treated with cyclophosphamide, assuming equivalent therapeutic effects to RTX.

Statistical significance was defined as a two-sided *p*-value < 0.05. All statistical analyses were performed using the R software (version 4.2.2).

## Results

### Study population and background characteristics

**Fig. 1:**
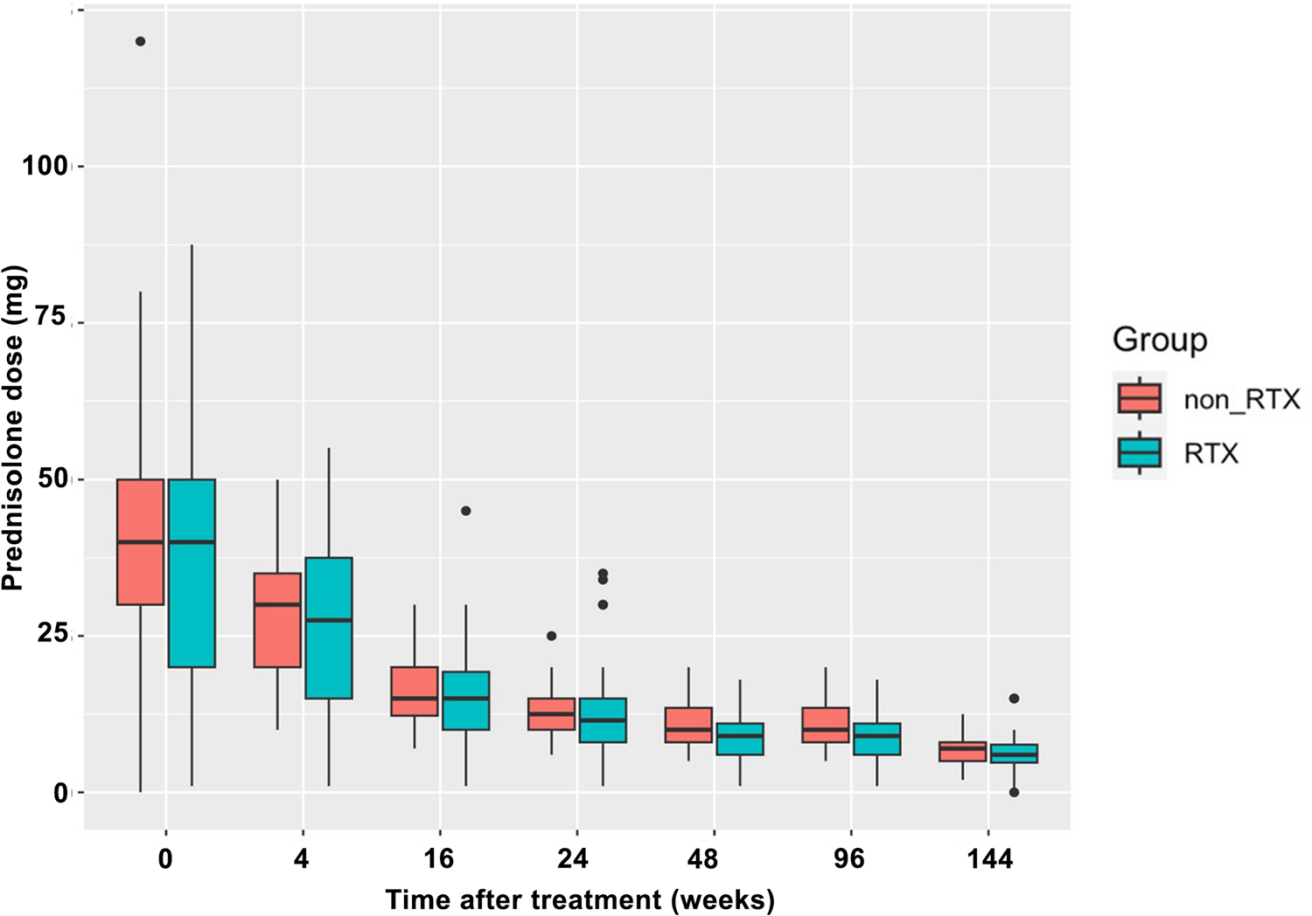
Prednisolone dosage at different time points. This box-and-whisker plot diagram illustrates the prednisolone dosage up to 144 weeks in the RTX and non-RTX groups. RTX, rituximab

One hundred patients were included in the analysis; Table 1 shows the baseline characteristics of the participants. Details of organ involvement are provided in Supplementary Table 1. The RTX group had a higher prevalence of GPA and PR3-ANCA. Furthermore, the BVAS at relapse was not significantly different between the two groups (9 vs. 10 points). The dosage of glucocorticoids (prednisolone equivalent) administered was similar in both groups (Fig. 1). For induction therapy, RTX was administered at a standard dose of 375 mg/m^2^, with patients receiving between one and four doses: one dose in five patients, two in six, three in seven and four in 34. RTX for maintenance was administered to 30 of 52 patients 6 months after induction at doses of 500 mg/body or 375 mg/m^2^.

**Table 1:** Baseline characteristics of the enrolled patients

	Overall (*n* = 100)	RTX-group (*n* = 52)	Non-RTX group (*n* = 48)	*p*-value
Age (years), median (IQR)	72 (64–79)	70.5 (57.2–75)	74.5 (69–81)	0.004*
Sex (female), *n* (%)	53 (53)	25 (48)	28 (58)	0.304
AAV subtype, *n* (%)				0.002*
GPA	47 (47)	32 (62)	15 (31)	
MPA	53 (53)	20 (38)	33 (68)	
ANCA serotype, *n* (%)				0.023*
PR3-ANCA	32 (32)	23 (44)	9 (19)	
MPO-ANCA	65 (65)	28 (54)	37 (77)	
Both negative	3 (3)	1 (1.9)	2 (4.1)	
BVAS at relapse, median (IQR)	9.5 (6.0–13.75)	9 (6–12.7)	10 (6–14.75)	0.597
Organ involvement, *n* (%)				
General	26 (26)	13 (25)	13 (27)	0.812
Cutaneous	9 (9)	6 (12)	3 (6.3)	0.360
Mucous membranes/eyes	15 (15)	9 (17)	6 (13)	0.504
Ear, nose and throat	23 (23)	17 (33)	6 (13)	0.017*
Cardiovascular	1 (1)	0 (0.0)	1 (2.1)	0.301
Gastrointestinal	1 (1)	1 (1.9)	0 (0)	0.330
Pulmonary	26 (26)	18 (35)	8 (16)	0.041*
Renal	50 (50)	21 (40)	29 (60)	0.046*
Nervous system	35 (35)	24 (46)	11 (23)	0.015*
Glucocorticoid dose before relapse, mg/day (PSL equivalent), median(IQR)	9 (5–13.5)	9 (5–11)	9.5 (5–15)	0.437
Immunosuppressants used before and after relapse, *n* (%)				
Before relapse				
Rituximab	5 (5)	4 (7.7)	1 (2.0)	0.199
Cyclophosphamide	8 (8)	6 (12)	2 (4.2)	0.174
Mycophenolate mofetil	6 (6)	4 (7.6)	2 (4.2)	0.458
Azathioprine	25 (25)	16 (31)	9 (19)	0.166
Methotrexate	8 (8)	7 (13)	1 (2.0)	0.036*
Mizoribine	8 (8)	3 (5.8)	5 (10)	0.392
After relapse				
Glucocorticoid dose after relapse, mg/day (PSL equivalent), median(IQR)	40 (27–50)	40 (20–50)	40 (30–50)	
Cyclophosphamide	20 (20)	4 (7.7)	16 (33)	0.001*
Cumulative dose, mg, median (IQR)	1.7 (1–2.6)	1.7 (1.4–2.5)	1.9 (1–2.6)	NA
Mycophenolate mofetil	7 (7)	4 (7.7)	3 (6.3)	0.778
Azathioprine	24 (24)	8 (15)	16 (33)	0.036*
Methotrexate	7 (7)	6 (12)	1 (2.1)	0.064
Mizoribine	5 (5)	1 (1.9)	4 (8.3)	0.142

Data are presented as median and interquartile range or *n* (%)

Abbreviations: *RTX* rituximab, *AAV* anti-neutrophil cytoplasmic antibody associated vasculitis, *GPA* granulomatosis with polyangiitis, *MPA* microscopic polyangiitis, *PR3* proteinase 3, *MPO* myeloperoxidase, *BVAS* Birmingham Vasculitis Activity Score, *PSL* prednisolone, *NA* not applicable

^*^ < 0.05

### CR at week 24

**Fig. 2:**
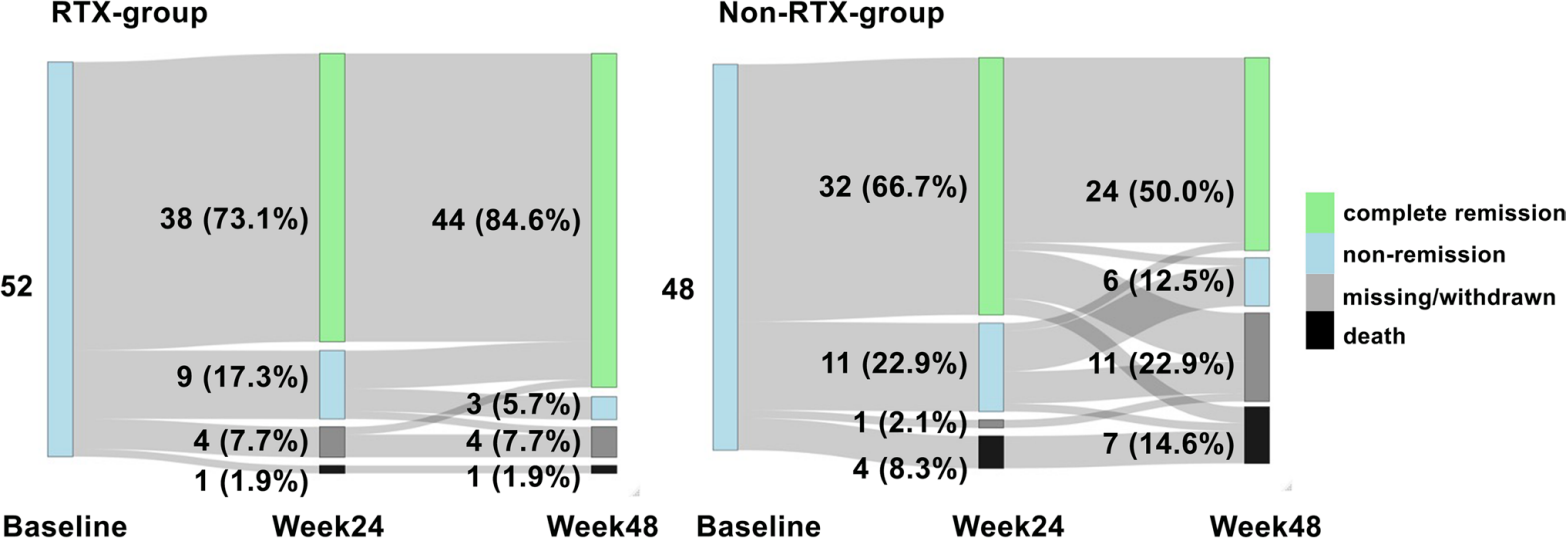
Sankey diagram illustrating proportions of complete remission, non-complete remission, missing/withdrawal and death at baseline, 24 weeks and 48 weeks in the RTX (left) and non-RTX groups (right). Patients who achieved CR are classified as “complete remission”, those who did not achieve CR as “non-remission”, those with missing data or who withdrew from the registry as “missing/withdrawn” and those who passed away as “death”. The values in the figure represent the number of individuals and their proportions at each time point. RTX, rituximab; CR, complete remission

The proportion of patients who achieved CR at week 24 was higher in the RTX group than in the non-RTX group; however, the difference was not statistically significant (38 [79.2%] vs. 32 [68.1%], *p* = 0.321) (Fig. 2; Table 2). The adjusted odds ratios for achieving CR at 24 weeks with the use of RTX after multiple imputations were 1.27 (95% confidence interval [CI] 0.47–3.51) (Table 3).

**Table 2:** Summary of the efficacy and safety outcome in complete cases

	RTX group (*n* = 52)	Non-RTX group (*n* = 48)	*p*-value
Complete remission at week 24, n (%)	38/48 (79.2)	32/47 (68.1)	0.321
BVAS at week 24, median (IQR)^†^	0 (0–2.25)	2 (0–5.0)	0.058
Complete remission at week 48, *n* (%)	44/48 (91.7)	24/37 (64.9)	0.005*
BVAS at week 48, median (IQR)^‡^	0 (0–2.0)	0 (0–3.0)	0.065
Serious infection up to week 48, *n* (%)	8 (15.4)	12 (25.0)	0.342

Data are presented as median and interquartile range or *n* (%)

Abbreviations: *RTX* rituximab, *CR* complete remission, *BVAS* Birmingham Vasculitis Activity Score

^*^ < 0.05

^†^Outcome reported for RTX group (*n* = 49) and non-RTX group (*n* = 47)

^‡^Outcome reported for RTX group (*n* = 48) and non-RTX group (*n* = 39)

**Table 3:** Association between RTX use and complete remission at weeks 24 and 48 after multiple imputation

	Odds ratio (95% CI)			
	Unadjusted	*p*-value	Adjusted	*p*-value
Week 24				
RTX use	1.52 (0.63–3.74)	0.359	1.27 (0.47–3.51)	0.636
Week 48				
RTX use	3.83 (1.41–11.67)	0.011	2.95 (0.97–9.91)	0.065

Unadjusted and adjusted logistic regression analysis, considering age, ANCA serotype, AAV subtype and prednisolone dosage at re-induction treatment as covariates

Abbreviations: *CI* confidence interval, *RTX* rituximab

### CR at week 48 and the incidence of severe infection

At week 48, the RTX group had a significantly higher proportion of patients who achieved CR (*p* = 0.005) (Table 2). The adjusted odds ratio for achieving CR at 48 weeks with the use of RTX after multiple imputations was 2.95 (95% CI 0.97–9.91) (Table 3). Regarding safety, the incidence of severe infections tended to be lower in the RTX group than in the non-RTX group [8 (15.4%) vs. 12 (25.0%), *p* = 0.342] (Table 2).

### Subgroup analyses by ANCA serotype and AAV subtype

**Fig. 3:**
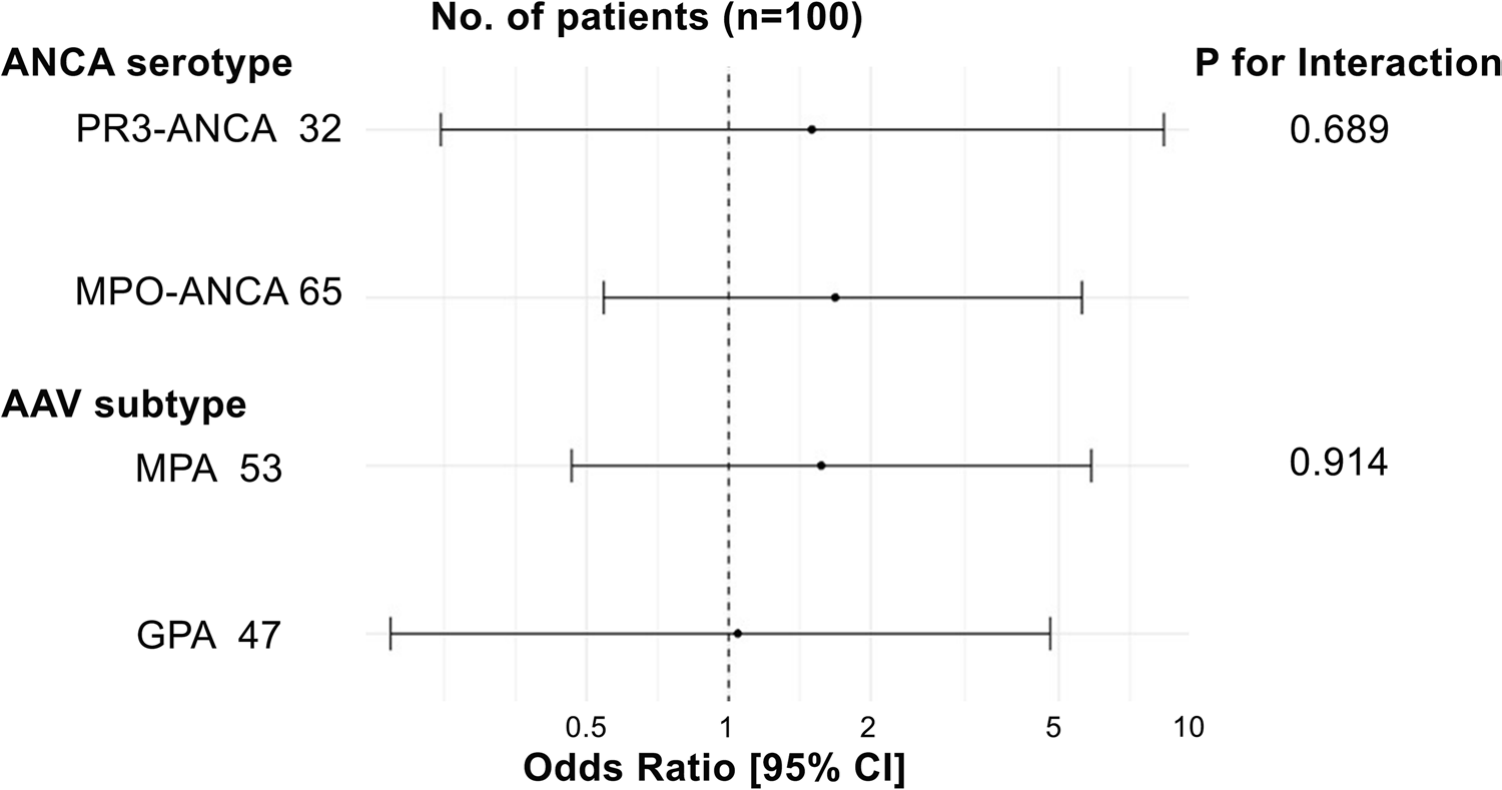
Subgroup analysis by ANCA serotype and AAV subtype on the association between RTX use and complete remission. The forest plot describes the results of the subgroup analysis by ANCA serotype and AAV subtype on the association between RTX use and complete remission. We depict the odds ratio for achieving remission with RTX use. ANCA, antineutrophil cytoplasmic antibody; AAV, ANCA-associated vasculitis; RTX, rituximab

Subgroup analyses to assess the interactions of RTX with ANCA serotype and AAV subtype showed no apparent qualitative interaction in RTX effect (Fig. 3).

### Sensitivity analysis

Using 1:1 propensity score matching, 33 patients from the RTX and non-RTX groups were identified. No significant differences in background characteristics were observed between the two groups (Supplementary Table 2). The odds ratio for achieving remission in the RTX group was 1.89 (95% CI 0.63–5.99) at 24 weeks and 5.2 (95% CI 1.32–26.28) at 48 weeks.

In the complete-case analysis, the odds ratio for achieving remission was 1.54 (95% CI 0.48–5.21) at 24 weeks and 4.10 (95% CI 0.82–25.91) at 48 weeks in multivariate analysis. This result was consistent with the result of the main analysis (Supplementary Table 3).

In the non-RTX group, 16 individuals who received cyclophosphamide, a treatment considered to be as effective as RTX in previous studies, were compared with the RTX group. The baseline characteristics of the patients are described in Supplementary Table 4. Cyclophosphamide was administered intravenously in all but one patient, with a frequency of administration of 1–6. The cumulative dose is detailed in Table 1. A trend toward higher BVAS was observed in cyclophosphamide-treated patients. The proportion of patients achieving CR at week 24 in the RTX- and cyclophosphamide-treated groups was similar (38 [79.2% vs. 12 [75.0%], *p* = 1.000]) (Supplementary Table 5). However, at week 48, the RTX group had a significantly higher proportion of patients achieving CR.

## Discussion

This study explored the effectiveness and safety of RTX in patients with severely relapsed AAV using a multicentre registry database. We found numerically higher CR proportions at weeks 24 and 48 in the RTX group than in the non-RTX group with tolerable safety; however, there was no statistically significant difference in odds ratios at week 24 and the incidence of serious infection for RTX treatment after adjusting for multiple factors.

Previous studies confirmed the effectiveness of RTX in AAV relapse [10, 12]. A recent study including patients from the RITAZERAM trial showed a 90% remission rate after 4 months of RTX treatment in patients who experienced a relapse [10]. In our study, the CR rate in the RTX group was 90.0% at 48 weeks, which is consistent with the results of previous studies. B cell repopulation within 1 year of B cell depletion therapy is known to increase the risk of AAV relapse [17]. Furthermore, relapses after RTX treatment are associated with low plasma RTX concentration [19]. Therefore, our findings are consistent with this pathophysiological understanding.

This study has three strengths compared with previous studies. First, our study analysed outcomes at 24 and 48 weeks, providing a more extended observation period. This longer observation period effectively demonstrated the long-term effectiveness of RTX treatment. Second, we focused on patients experiencing severe relapses with a higher median BVAS than those in previous studies. Because RTX is typically recommended for severe cases, our study provides valuable insights into this specific patient group [15]. Third, we obtained detailed data on disease activity, laboratory data and treatment drugs from our registry compared to a previous study [13]. Our data showed that the incidence of serious infections did not increase in patients receiving RTX, suggesting a potential risk–benefit advantage. Therefore, we believe that our results will provide useful information for physicians to determine an appropriate treatment strategy for relapsed AAV cases.

The present study has several limitations. First, it was a multicentre, retrospective study in which patient treatment was guided by the treating physician’s judgment, which could have led to confounding by indication. The RTX group demonstrated a higher prevalence of PR3-ANCA and GPA, with notable pulmonary and nerve involvements, whereas the non-RTX group exhibited a greater incidence of renal involvement. It is unknown which specific organ involvement responds best to RTX treatment; however, the differences in organ involvement between the groups might have contributed to the observed differences in outcomes. However, we observed improved therapeutic outcomes in the RTX group, even with a higher proportion of patients with PR3-ANCA and GPA. Second, the relatively small sample size may have resulted in the lack of statistically significant differences in certain outcomes. Despite the limited data, this study indicates a positive effect of RTX on relapsed AAV over a prolonged observation period. The larger the sample size, the more likely the detection of statistical differences. Third, although our study only included patients who experienced severe relapse, many of those who did not receive RTX treatment did not receive cyclophosphamide recommended for severe relapse. This may have led to suboptimal treatment in the control group, potentially overestimating the effectiveness of RTX treatment. Consequently, we compared the outcomes between the RTX- and cyclophosphamide-treated groups. We found that the proportion of patients with CR was higher in the RTX group, which is consistent with our main analysis. Finally, because this was a retrospective study, we could not standardize the frequency of RTX administration; five patients received one dose of RTX and six received two doses. The typical dose in Japan is 375 mg/m^2^ of body surface area. The inclusion of patients who received one or two doses of RTX might have led to an underestimation of the therapeutic effect owing to suboptimal treatment intensity. The effectiveness of RTX even under these conditions validates the findings of this study.

This study revealed the potential effectiveness and safety of RTX compared with those of traditional immunosuppressive therapy in patients with severely relapsed AAV. Although no statistically significant difference was observed at 24 weeks, clinicians may safely induce CR in a larger number of patients using RTX, irrespective of the ANCA serotype or AAV subtype. Thus, further prospective, large-scale studies are warranted.

## Supplementary Information

Below is the link to the electronic supplementary material.

Supplementary file1 (DOCX 52 KB)

## Data Availability

The datasets generated during and/or analysed during the current study are available from the corresponding author on reasonable request.
