# Supplementary material for: Effectiveness and safety of rituximab in severely relapsed antineutrophil cytoplasmic antibody–associated vasculitis: a retrospective analysis of a Japanese multicentre cohort from the J-CANVAS
Source: Clin Rheumatol. 2024 Aug 12;43(10):3195–204. doi: 10.1007/s10067-024-07096-y (PMC11442524; doi:10.1007/s10067-024-07096-y)
Supplement: Supplementary file 1 — Supplementary file1 (DOCX 52 KB) [file 10067_2024_7096_MOESM1_ESM.docx]

**Supplemental material**

Effectiveness and safety of rituximab in severely relapsed antineutrophil cytoplasmic antibody-associated vasculitis: a retrospective analysis of a Japanese multicenter cohort from the J-CANVAS

Authors: Genki Kidoguchi, Yusuke Yoshida, Hirofumi Watanabe, Tomohiro Sugimoto, Sho Mokuda^,^ Takashi Kida, Nobuyuki Yajima, Satoshi Omura, Daiki Nakagomi, Yoshiyuki Abe, Masatoshi Kadoya, Naoho Takizawa, Atsushi Nomura, Yuji Kukida, Naoya Kondo, Yasuhiko Yamano, Takuya Yanagida, Koji Endo, Kiyoshi Matsui, Tohru Takeuchi, Kunihiro Ichinose, Masaru Kato, Ryo Yanai, Yusuke Matsuo, Yasuhiro Shimojima, Ryo Nishioka, Ryota Okazaki, Tomoaki Takata, Takafumi Ito, Mayuko Moriyama, Ayuko Takatani, Yoshia Miyawaki, Toshiko Ito-Ihara, Takashi Kawaguchi, Yutaka Kawahito, and Shintaro Hirata, on behalf of Japan Collaborative Registry of ANCA-Associated Vasculitis (J-CANVAS)

*Corresponding Author:

Genki Kidoguchi, M.D.

Department of Clinical Immunology and Rheumatology, Hiroshima University Hospital

E-mail: [kidogen@hiroshima-u.ac.jp](mailto:kidogen@hiroshima-u.ac.jp)

**Supplemental Table 1**

Details of organ involvement at relapse

| Organ involvement, n | All cases  (n=100) |
| --- | --- |
| 1. General | 26 |
| Myalgia | 9 |
| Arthralgia/Arthritis | 7 |
| Fever≧38℃ | 12 |
| Weight loss≧2kg | 6 |
| 2. Cutaneous | 9 |
| Infarct | 1 |
| Purpura | 6 |
| Ulcer | 2 |
| Gangrene | 0 |
| Other skin vasculitis | 1 |
| 3. Mucous membranes/eyes | 15 |
| Mouth ulcers | 1 |
| Genital ulcers | 0 |
| Adnexal inflammation | 0 |
| Significant proptosis | 3 |
| Scleritis/Episcleritis | 5 |
| Conjunctivitis / Blepharitis / Keratitis | 0 |
| Blurred vision | 4 |
| Sudden visual loss | 7 |
| Uveitis | 0 |
| Retinal changes (vasculitis/thrombosis/exudate/haemorrhage) | 1 |
| 4. Ear, nose, and throat | 23 |
| Bloody nasal discharge/crusts/ulcers/granulomata | 7 |
| Paranasal sinus involvement | 17 |
| Subglottic stenosis | 1 |
| Conductive hearing loss | 5 |
| Sensorineural hearing loss | 7 |
| 5. Pulmonary | 26 |
| Wheeze | 2 |
| Nodules or cavities | 17 |
| Pleural effusion/pleurisy | 5 |
| Infiltrate | 19 |
| Endobronchial involvement | 0 |
| Massive haemoptysis/alveolar haemorrhage | 10 |
| Respiratory failure | 4 |
| 6. Cardiovascular | 1 |
| Loss of pulses | 0 |
| Valvular heart disease | 1 |
| Pericarditis | 0 |
| Ischaemic cardiac pain | 1 |
| Cardiomyopathy | 0 |
| Congestive heart failure | 1 |
| 7. Abdominal | 1 |
| Peritonitis | 1 |
| Bloody diarrhoea | 0 |
| Ischaemic abdominal pain | 0 |
| 8. Renal | 50 |
| Hypertension | 3 |
| Proteinuria > 1+ | 39 |
| Haematuria ≧ 10RBCs/hpf | 37 |
| Creatinine 125-249 μ/L (1.41-2.82mg/dl) | 14 |
| Creatinine 250-499 μ/L (2.83-5.66mg/dl) | 11 |
| Creatinine ≧ 500 μ/L (≧ 5.66mg/dl) | 2 |
| Rise in serum creatinine > 30% or fall in creatinine clearance > 25% | 15 |
| 9. Nervous system | 35 |
| Headache | 19 |
| Meningitis | 3 |
| Organic confusion | 0 |
| Seizures (not hypertensive) | 2 |
| Cerebrovascular accident | 0 |
| Spinal cord lesion | 0 |
| Cranial nerve palsy | 9 |
| Sensory peripheral neuropathy | 9 |
| Mononeuritis multiplex | 5 |
| Data are presented as n. | |

**Supplementary Table 2**

Baseline characteristics of the enrolled patients Before and After propensity score matching

|  | Before propensity score matching | |  | After propensity score matching | |  |  |
| --- | --- | --- | --- | --- | --- | --- | --- |
|  | RTX　group(n=52) | non-RTX group(n=48) | p-  value | RTX-group(n=33) | non-RTX group(n=33) | p-value | SMD |
| Age(years), median(IQR) | 70.5(57-75) | 74.5(69-81) | 0.004 | 71.0(66-79) | 72(69-79) | 0.488 |  |
| Sex(female), n(%) | 25(48) | 28(58) | 0.304 | 14(42) | 19(58) | 0.325 | 0.307 |
| AAV subtype, n(%) |  |  | 0.002 |  |  | 1 | <0.001 |
| GPA | 32(62) | 15(31) |  | 13(39) | 13(39) |  |  |
| MPA | 20(38) | 33(68) |  | 20(61) | 20(61) |  |  |
| ANCA serotype, n(%) |  |  | 0.023 |  |  | 0.838 | 0.147 |
| PR3-ANCA | 23(44) | 9(19) |  | 8(24.2) | 8(24.2) |  |  |
| MPO-ANCA | 28(54) | 37(77) |  | 24(73) | 23(70) |  |  |
| Both negative | 1(1.9) | 2(4.1) |  | 1(3) | 2(6) |  |  |
| BVAS at replase, median(IQR) | 9(6-12.7) | 10(6-14.75) | 0.597 | 10(6-13) | 10(7-14) | 0.598 | 0.103 |
| Glucocorticoid dose after relapse,mg/day (PSL equivalent), median(IQR) | 40(20-50) | 40(30-50) | 0.437 | 40(30-50) | 40(30-50) | 0.670 | 0.031 |
| Data are presented as median and interquartile range or n(%).  Abbreviations: RTX, rituximab; SMD; standardized mean difference; AAV, anti-neutrophil cytoplasmic antibody associated vasculitis; GPA, granulomatosis with polyangiitis; MPA, microscopic polyangiitis; PR3, proteinase 3; MPO, myeloperoxidase; BVAS, Birmingham Vasculitis Activity Score. PSL, prednisolone | | | | | | | |

**Supplementary Table 3**

Association between RTX use and complete remission at week24 and 48 in complete-case analysis

|  | Odds Ratio (95%CI) | | | |
| --- | --- | --- | --- | --- |
|  | Univariate | P-value | Multivariate | P-value |
| Week24 |  |  |  |  |
| RTX use | 1.45 (0.54-4.02) | 0.465 | 1.54(0.48-5.21) | 0.471 |
| Week48 |  |  |  |  |
| RTX use | 3.67(0.89-18.62) | 0.084 | 4.10(0.82-25.91) | 0.099 |
| Unadjusted and adjusted logistic regression analysis, considering age, ANCA serotype, AAV subtype, and PSL dosage at re-induction treatment as covariates.  Abbreviations; CI, confidence interval; RTX, rituximab. | | | | |

**Supplementary Table 4**

Baseline characteristics of the RTX group and CY group

|  | RTX-group (n=52) | CY-group (n=16) | p-value |
| --- | --- | --- | --- |
| Age(years), median(IQR) | 70.5(57.2-75) | 72.0(69.8-76.3) | 0.373 |
| Sex(female), n(%) | 25(48) | 10(63) | 0.469 |
| AAV subtype, n(%) |  |  | 0.160 |
| GPA | 32(62) | 6(37) |  |
| MPA | 20(38) | 10(63) |  |
| ANCA serotype, n(%) |  |  | 0.305 |
| PR3-ANCA | 23(44) | 4(25) |  |
| MPO-ANCA | 28(54) | 12(75) |  |
| Both negative | 1(1.9) | 0 |  |
| BVAS at relapse, median(IQR) | 9(6-12.7) | 13.5(8.5-17.3) | 0.040^*^ |
| Organ involvement, n(%) |  |  |  |
| General | 13(25) | 5(31) | 0.864 |
| Cutaneous | 6(12) | 2(13) | 1.000 |
| Mucous membranes/eyes | 9(17) | 2(13) | 0.945 |
| Ear, nose, and throat | 17(33) | 3(19) | 0.449 |
| Cardiovascular | 0(0.0) | 1(6) | 0.530 |
| Gastrointestinal | 1(1.9) | 0(0.0) | 1.000 |
| Pulmonary | 18(35) | 9(56) | 0.210 |
| Renal | 21(40) | 9(56) | 0.407 |
| Nervous system | 24(46) | 6(38) | 0.748 |
| Glucocorticoid dose before relapse (PSL equivalent), median(IQR) | 9(5-11) | 11(5-19) | 0.299 |
| Immunosuppressants used before and after relapse, n(%) | | | |
| Before relapse |  |  |  |
| Rituximab | 4(7.7) | 0(0.0) | 0.592 |
| Cyclophosphamide | 6(12) | 1(6.2) | 0.890 |
| Mycophenolate mofetil | 4(7.6) | 0(0.0) | 0.592 |
| Azathioprine | 16(31) | 2(11) | 0.261 |
| Methotrexate | 7(13) | 1(6.2) | 0.734 |
| Mizoribine | 3(5.8) | 2(13) | 0.723 |
| After relapse |  |  |  |
| Glucocorticoid dose after relapse ,mg/day (PSL equivalent), median(IQR) | 40(20-50) | 45(34-53) | 0.428 |
| Mycophenolate mofetil | 4(7.7) | 0(0.0) | 0.592 |
| Azathioprine | 8(15) | 5(31) | 0.295 |
| Methotrexate | 6(12) | 1(6) | 0.890 |
| Mizoribine | 1(1.9) | 1(6) | 0.960 |
| ^*^<0.05  Data are presented as median and interquartile range or n(%).  Abbreviations: RTX, rituximab; CY, cyclophosphamide; AAV, anti-neutrophil cytoplasmic antibody associated vasculitis; GPA, granulomatosis with polyangiitis; MPA, microscopic polyangiitis; PR3, proteinase 3; MPO, myeloperoxidase; BVAS, Birmingham Vasculitis Activity Score; PSL, prednisolone. | | | |

**Supplemental Table 5**

Association between complete remission and treatment in complete cases

|  | RTX-group  (n=52) | CY-group  (n=16) | p-value |
| --- | --- | --- | --- |
| Complete remission at week 24, n(%)† | 38(79.2) | 12(75.0) | 1.000 |
| Complete remission at week 48, n(%)† | 44(91.7) | 10(62.5) | 0.017^*^ |
| ^*^<0.05  Data are presented as n(%).  Abbreviations: RTX, rituximab; CY, cyclophosphamide.  †Outcome reported for RTX group (n=48) and CY group (n=16) | | | |

**Supplemental Table 6**

Names of participating institutions, names of the corresponding institutional review board (IRB), and their approval dates

| Institution Name | Ethics approval dates | IRB name |
| --- | --- | --- |
| Kyoto Prefectural University of Medicine | 25 January 2021 | Ethics Committee, Kyoto Prefectural University of Medicine |
| University of Yamanashi Hospital | 30 March 2021 | Ethical Review Committee of the University of Yamanashi Hospital |
| Juntendo University | 3 September 2021 | Juntendo University Hospital IRB |
| Japanese Red Cross Society Kyoto Daiichi Hospital | 11 March 2021 | Ethical Committees of the Japanese Red Cross Society Kyoto Daiichi Hospital |
| Chubu Rosai Hospital | 23 March 2021 | Chubu Rosai Hospital Institutional Review Board |
| St. Luke's International Hospital | 9 April 2021 | Institutional review board of St. Luke's International Hospital |
| Japanese Red Cross Society Kyoto Daini Hospital | 10 August 2021 | Ethics Committee of the Japanese Red Cross Kyoto Daini Hospital |
| Kyoto Katsura Hospital | 7 May 2021 | Ethics Committee of the Kyoto Katsura Hospital |
| Tosei General Hospital | 28 May 2021 | Ethics Committee of Tosei General Hospital |
| Kagoshima University Hospital | 25 June 2021 | Kagoshima University Hospital Institutional Review Board |
| Tottori Prefectural Central Hospital | 21 May 2021 | Ethics Committee of Tottori Prefectural Central Hospital |
| Hiroshima University Hospital | 2 June 2021 | Ethical Committee for Epidemiology of Hiroshima University |
| Hyogo Medical University, School of Medicine | 24 May 2021 | Ethics Committee of Hyogo Medical University Hospital |
| Osaka Medical and Pharmaceutical University | 29 July 2021 | Osaka Medical and Pharmaceutical University Ethics Committee |
| Nagasaki University Graduate School of Biomedical Sciences | 12 July 2021 | Ethics Committee of the Nagasaki University Graduate School of Biomedical Sciences |
| Hokkaido University | 26 July 2021 | Institutional review board of the Hokkaido University Hospita |
| Showa University School of Medicine | 11 June 2021 | Ethical committees of the Showa University Hospital |
| Tokyo Kyosai Hospital | 23 April 2021 | Ethics Committee of Tokyo Kyosai Hospital |
| Shinshu University School of Medicine | 27 April 2021 | local ethics committeeof Shinshu University |
| Kanazawa University | 7 July 2021 | Ethics Committee of Kanazawa University Hospital |
| Tottori University | 3 August 2021 | Ethics Committee of the Tottori University Faculty of Medicine |
| Shimane University Faculty of Medicine | 21 May 2021 | Shimane University Institutional Committee on Ethics |
| Sasebo Chuo Hospital | 14 June 2021 | Ethics Committee of Sasebo Chuo Hospital |
| Okayama University Graduate School of Medicine, Dentistry and Pharmaceutical Sciences | 25 June 2021 | Okayama University Ethics Committee |
